# Epstein-Barr Virus-Encoded LMP2A Induces an Epithelial–Mesenchymal Transition and Increases the Number of Side Population Stem-like Cancer Cells in Nasopharyngeal Carcinoma

**DOI:** 10.1371/journal.ppat.1000940

**Published:** 2010-06-03

**Authors:** Qing-Li Kong, Li-Juan Hu, Jing-Yan Cao, Yi-Jun Huang, Li-Hua Xu, Yi Liang, Dan Xiong, Su Guan, Bao-Hong Guo, Hai-Qiang Mai, Qiu-Yan Chen, Xing Zhang, Man-Zhi Li, Jian-Yong Shao, Chao-Nan Qian, Yun-Fei Xia, Li-Bing Song, Yi-Xin Zeng, Mu-Sheng Zeng

**Affiliations:** 1 State Key Laboratory of Oncology in South China, Sun Yat-Sen University Cancer Center, Guangzhou, People's Republic of China; 2 Department of Experimental Research, Sun Yat-Sen University Cancer Center, Guangzhou, People's Republic of China; 3 Affiliated Hospital of Jining Medical College, Shandong, People's Republic of China; 4 Zhongshan School of Medicine, Sun Yat-Sen University, Guangzhou, People's Republic of China; 5 Department of Nasopharyngeal Carcinoma, Sun Yat-Sen University Cancer Center, Guangzhou, People's Republic of China; 6 Department of Radiotherapy, Sun Yat-Sen University Cancer Center, Guangzhou, People's Republic of China; University of Wisconsin-Madison, United States of America

## Abstract

It has been recently reported that a side population of cells in nasopharyngeal carcinoma (NPC) displayed characteristics of stem-like cancer cells. However, the molecular mechanisms underlying the modulation of such stem-like cell populations in NPC remain unclear. Epstein-Barr virus was the first identified human tumor virus to be associated with various malignancies, most notably NPC. LMP2A, the Epstein-Barr virus encoded latent protein, has been reported to play roles in oncogenic processes. We report by immunostaining in our current study that LMP2A is overexpressed in 57.6% of the nasopharyngeal carcinoma tumors sampled and is mainly localized at the tumor invasive front. We found also in NPC cells that the exogenous expression of LMP2A greatly increases their invasive/migratory ability, induces epithelial–mesenchymal transition (EMT)-like cellular marker alterations, and stimulates stem cell side populations and the expression of stem cell markers. In addition, LMP2A enhances the transforming ability of cancer cells in both colony formation and soft agar assays, as well as the self-renewal ability of stem-like cancer cells in a spherical culture assay. Additionally, LMP2A increases the number of cancer initiating cells in a xenograft tumor formation assay. More importantly, the endogenous expression of LMP2A positively correlates with the expression of ABCG2 in NPC samples. Finally, we demonstrate that Akt inhibitor (V) greatly decreases the size of the stem cell side populations in LMP2A-expressing cells. Taken together, our data indicate that LMP2A induces EMT and stem-like cell self-renewal in NPC, suggesting a novel mechanism by which Epstein-Barr virus induces the initiation, metastasis and recurrence of NPC.

## Author Summary

Epstein-Barr virus (EBV) infects about 90% of people worldwide and persists benignly as a latent infection. However, EBV is associated with different types of human cancer. Nasopharyngeal carcinoma (NPC) is the most commonly known EBV associated cancer and expresses a well defined set of latent viral genes, including LMP2A, which has been detected in the majority of NPC samples. Several studies indicated this latent viral protein drove cellular invasion and metastasis. For this study, enforced LMP2A expressing NPC cell lines were generated. We show here that LMP2A induces an Epithelial–Mesenchymal Transition and increases the Stem-like Cancer Cells in NPC. Our results suggest that LMP2A supports tumor initiation and recurrence of the infected nasopharyngeal epithelial cells. For the first time we report a virus protein that functions in the initiation and progression of cancer by inducing the cancer stem-like cells. These findings permit a more detailed understanding of function and contribution to viral pathogenesis and provide a novel therapeutic target for NPC therapy.

## Introduction

Nasopharyngeal carcinoma (NPC) is the most frequent head and neck tumor in Guangdong, South China, where the incidence peaks at 50 per 100,000, but is rare in the Western world (1 per 100,000) [1], [2]. NPC is a highly malignant cancer which often invades adjacent regions and metastasizes to regional lymph nodes and distant organs. Thirty to 60 percent of patients with NPC will eventually develop a distant metastasis. Although NPC tumors are sensitive to radiotherapy and chemotherapy, treatment failure is high due to local recurrence and distant metastases, which are the key contributors to NPC mortality [3]. However, the underlying cellular and molecular mechanisms of NPC metastasis and recurrence remain poorly understood.

The epithelial–mesenchymal transition (EMT) is characterized as a switch from a polarized, epithelial phenotype to a highly motile fibroblastoid or mesenchymal phenotype. EMT is critical to metazoan embryogenesis, chronic inflammation and fibrosis, and has been demonstrated to be a central mechanism in cancer invasiveness and metastasis [4]. Recently, Weinberg and colleagues reported that EMT generates cells with stem cell-like properties [5], which suggests that metastases are sometimes caused by cancer cells that acquire stem cell characteristics. Recent studies have also suggested that cancer stem cells (CSCs) represent a small proportion of the cells in a tumor mass and contribute to tumor initiation, metastasis and recurrence. It has been further reported that cancer stem cells are enriched in side population (SP) cells which can efflux the DNA binding dye, Hoechst 33342, from the cell membrane [6], [7], [8]. Most recently, Wang and colleagues have reported that SP cells in the human NPC cell line CNE2 display stem cell characteristics [9]. However, the molecular mechanisms underlying the regulation of SP cells in NPC remain unclear.

Epstein-Barr virus (EBV), which ubiquitously infects more than 90% of the world's population, was the first human tumor virus identified to be causally associated with various lymphoid and epithelium malignancies [10]. However, the underlying mechanism of how EBV contributes to cancer is still poorly understood. NPC, particularly the undifferentiated type, is the most commonly known EBV associated cancer [11] and three EBV latent proteins are expressed in these tumors [12], [13]. EBNA1, whose primary role is to enable replication of the viral episomal genome [14], is the most widely expressed protein in NPC. However, although both LMP1 and LMP2A are detectable in NPC samples, much of the recent research focus has been on LMP1 because of its known oncogenic properties in B cells [15], [16]. However, LMP2A has been detected in more than 95% of NPC samples at the mRNA level, and about 50% of these specimens at protein level, whereas LMP1 could be detected in only about 65% or 35% of NPC samples at mRNA or protein level, respectively [17], [18], [19], [20], [21]. In addition, the high LMP2A expression in NPC samples has been reported to correlate with a poor survival outcome, although this study was carried out using only a small cohort [22].

Functional studies indicate that LMP2A plays an important role in the maintenance of EBV latent infection in B cells but is dispensable for EBV-driven B-cell transformation [23]. In epithelial cells, LMP2A has been reported to have transforming properties i.e. to alter cell motility and inhibit cell differentiation [22], [24], [25], [26]. Activation of the PI3K/Akt, NF-κB, β-catenin, STAT and Syk Tyrosine Kinase pathways has been suggested to contribute to the various functions of LMP2A in epithelial cells and B cells [26], [27], [28], [29], [30]. ITGα6 is thought to be involved in the enhancement of cell migration mediated by LMP2A [22]. Most recently, LMP2A has been reported to induce promoter hypermethylation of the *pten* gene in gastric carcinoma [31]. In addition, some of the above functions and pathways modulated by LMP2A have been reported to play roles in regulating the proliferation and self-renewal properties of CSCs [32], [33], [34]. These findings thus raise the possibility that LMP2A may affect oncogenic processes by modulating the CSC population in NPC.

We report in our current study that the stable expression of LMP2A in NPC cells induces cell invasion and EMT-like molecular alterations. More importantly, the overexpression of LMP2A increases the size of the stem-like cell population and the number of tumor initial cells. Our data thus represent the first indication that LMP2A has an effect on stem cell-like populations and provides additional clues to elucidating the role of LMP2A in NPC progression.

## Results

### Characterization of novel LMP2A monoclonal antibodies

**Figure 1:**
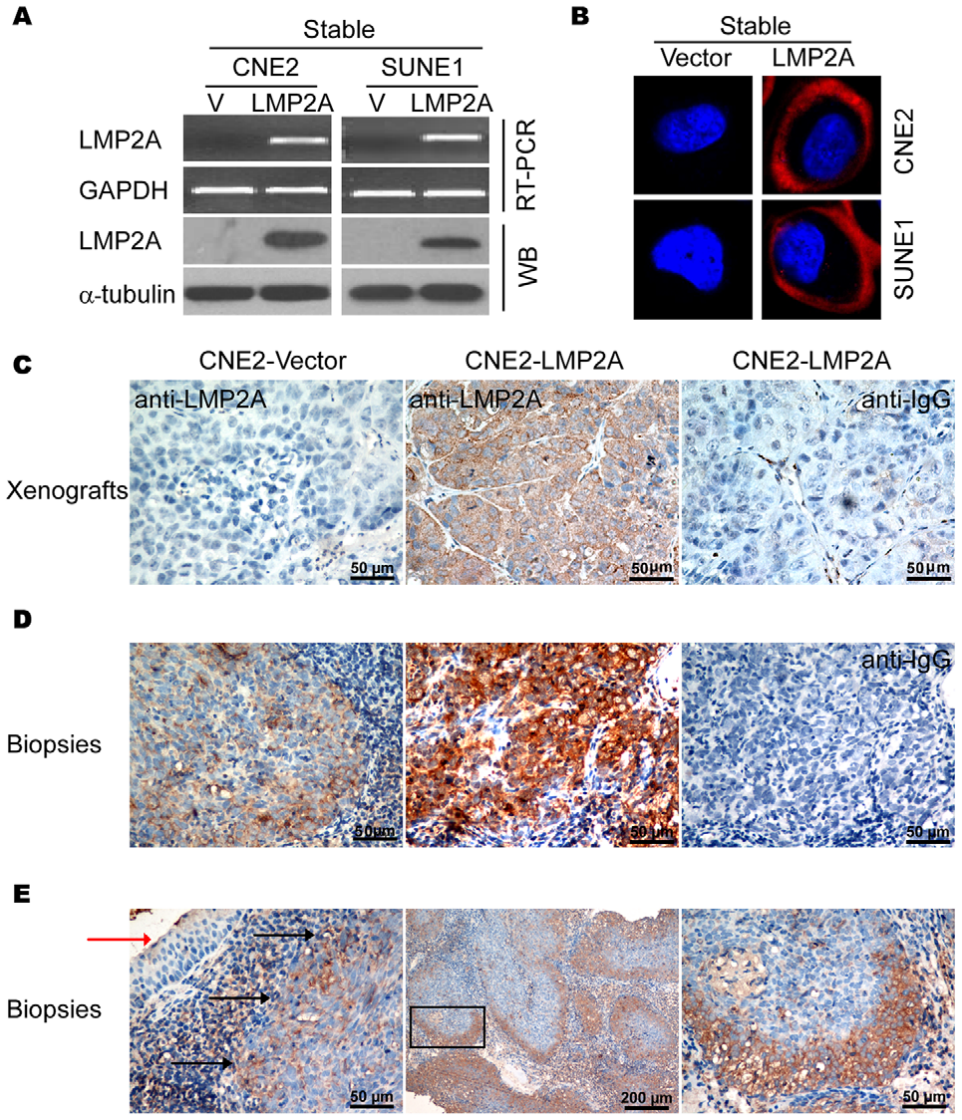
Endogenous and exogenous expression of LMP2A detected using monoclonal antibodies. A. Stable expression of LMP2A in NPC cells. The left and right panels reveal the stable ectogenic expression of LMP2A in both CNE2 and SUNE1 NPC cell lines. The upper and lower panels show mRNA and protein levels, respectively. B. Immunofluorescence staining clearly showing the membrane localization of LMP2A in the CNE2 and SUNE1 cell lines. C. Immunohistochemical analysis of LMP2A expression in a nude mice xenograft of LMP2A-expressing CNE2 cells using MoAb 4A11B3A3 or control IgG. D. Immunohistochemical analysis of endogenous LMP2A expression in NPC biopsies using MoAb 4A11B3A3 or control IgG. Representative moderate (left panel) and strong (middle panel) staining results are shown. E. Immunohistochemical analysis (left panel) with the MoAb 4A11B3A3 shows specific staining of LMP2A in NPC tumor nests (indicated by black arrows) but not in adjacent normal tissues (indicated by red arrow). The middle and right panels show that LMP2A is usually expressed in the invasive tumor front cells and is mainly localized on the cell membrane.

To detect LMP2A protein expression, monoclonal antibodies (MoAbs) was raised against a glutathione-S-transferase-fused full-length LMP2A protein (Proteintech Group Inc.). After primary selection by ELISA, five clones were obtained from the Proteintech Group and further characterized by western blotting and immunofluorescence staining. To establish stably expressed LMP2A cell lines, CNE2 and SUNE1 cells were infected with virus expressing either LMP2A in the pBabe vector or with empty vector alone, followed by selection in puromycin. No differences in the efficiency of selection between vector and LMP2A-infected cells were observed. RT-PCR analysis showed that LMP2A mRNA was expressed in both of the LMP2A-infected cell lines (Figure 1A). The expression of LMP2A protein was detectable by immunoblotting with four different LMP2A MoAb clones. Representative results for clone 4A11B3A3 are shown in Figure 1A. In contrast to LMP2A-infected cells, there was no detectable LMP2A mRNA or corresponding proteins in the vector control cells. The membrane localization of LMP2A in the LMP2A-infected NPC cells was confirmed by specific detection with 4A11B3A3 using immunofluorescence staining (Figure 1B).

To further determine whether MoAb 4A11B3A3 could detect LMP2A protein in archival NPC patient's biopsies, we tested this antibody in paraffin-embedded nude mice xenograft samples. In accordance with our western blot results, by immunohistochemical analysis we found strong membrane staining of LMP2A in CNE2-LMP2A inoculated samples. No specific staining was observed in CNE2-vector inoculated samples or in IgG detected controls (Figure 1C). We then analyzed endogenous LMP2A expression in NPC patient biopsies with the same MoAb by immunohistochemical analysis and obtained similar results (Figure 1D). Hence, the specificity and sensitivity of this antibody for endogenous and exogenous LMP2A expression by immunoblotting, immunohistochemistry and immunofluorescence analysis was verified.

### Expression of LMP2A in the invasive front of cancer nests in NPC biopsies

To further investigate the status of LMP2A expression in NPC biopsies, immunohistochemical analyses were carried out and revealed that 19 of 33 (57.6%) paraffin-embedded samples showed moderate (Figure 1D, right panel) to strong (Figure 1D, middle panel) staining of LMP2A in most of the tumor cells and in some scattered infiltrated lymphocytes. No positive staining was detected in adjacent noncancerous epithelial cells. As shown in Figure 1B and E, LMP2A is mainly expressed on the tumor cell membrane and preferentially located at the tumor invasive front. We then tested six archival relapse patient samples and found that were strongly positive for LMP2A expression. These data suggest that LMP2A is expressed in NPC samples at variable levels, that its localization at the invasive front is indicative of a potential role in promoting tumor invasion, and that the LMP2A protein levels may positively correlate with NPC recurrence.

### The exogenous expression of LMP2A in NPC cells induces EMT-like cellular marker alteration

**Figure 2:**
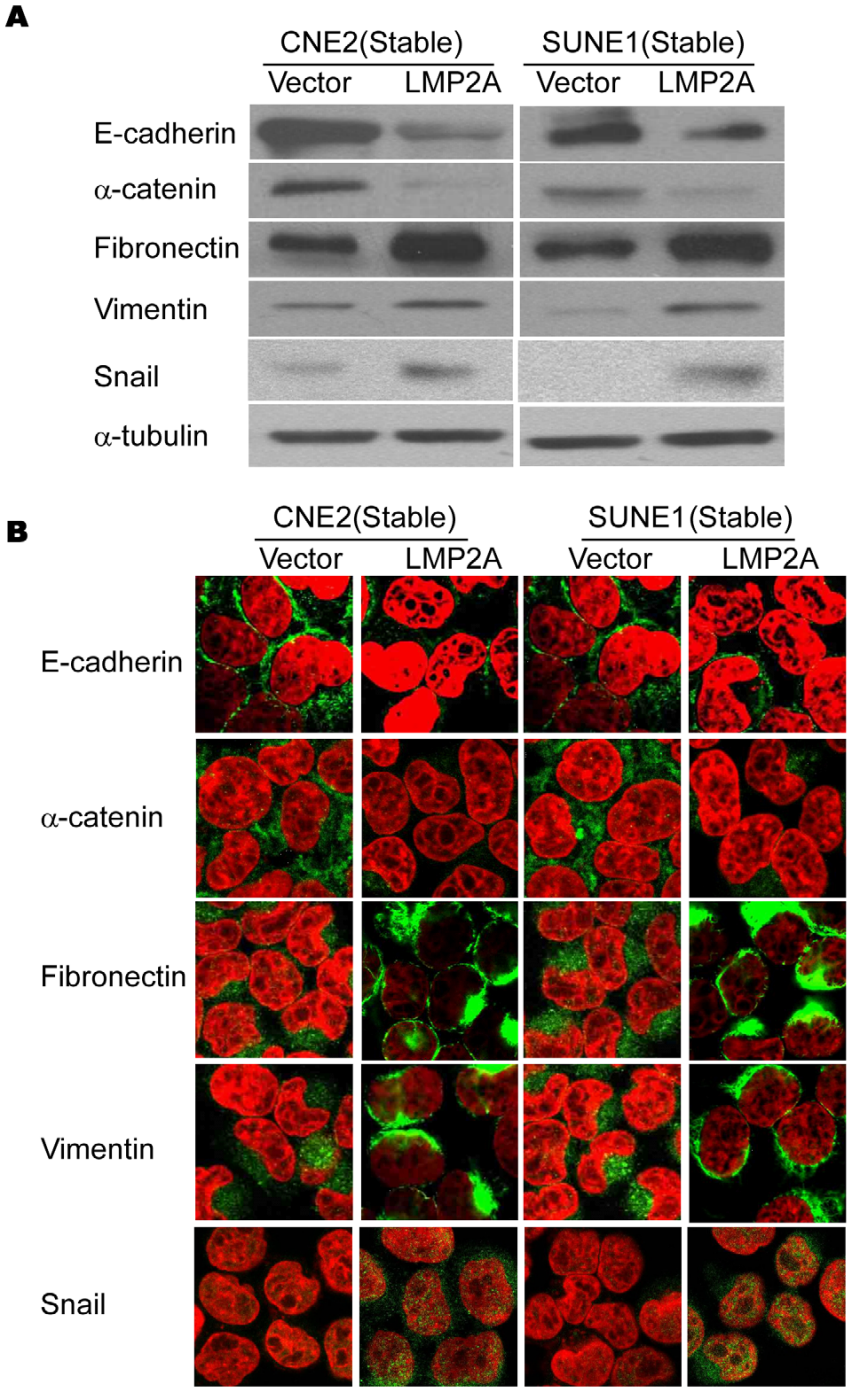
The overexpression of LMP2A in NPC cell lines induces EMT-like cellular marker alterations. A. Western blotting of E-cadherin, α-catenin, vimentin, fibronectin and snail in the vector control and LMP2A-expressing cells. B. Immunofluorescence staining reveals a reduced expression of E-cadherin, α-catenin and an increased expression of vimentin, fibronectin and snail in LMP2A-expressing cells in comparison with the vector controls.

It has been reported previously that LMP2A can promote the migratory/invasive properties of different epithelial cell types [35]. As determined by immunostaining, dissected tumor tissue samples from nude mice inoculated with CNE2-LMP2A cells showed a level of LMP2A that was comparable to that found in the NPC biopsies (Figure 1C and D). Hence, the established stable LMP2A expressing NPC cell line was found to contain physiological levels of LMP2A, and could thus be used in further studies of LMP2A function. Consistent with previous reports, the expression of LMP2A could enhance the migratory and invasive ability of NPC cells (data not show). Since the enhanced migratory/invasive ability of epithelial cells is often caused by EMT, we analyzed a panel of representative epithelial and mesenchymal markers by immunoblotting to determine whether this process occurs in LMP2A-expressing NPC cells. The results showed that the overexpression of LMP2A caused an EMT-like marker shift in the cells, including a dramatic downregulation of the epithelial markers E-cadherin and α-catenin, and upregulation of the mesenchymal markers fibronectin and the EMT-associated transcription factor snail, although the change of vimentin was moderate with about 2-fold increase in CNE2-LMP2A cells as analyzed by Quantity One software (Figure 2A). Immunofluorescence staining further revealed that the expression of E-cadherin and α-catenin, which shows membrane localization in control cells, dramatically decreased in LMP2A-expressing cells (Figure 2B, upper two panels). In contrast, the levels of fibronectin, vimentin and snail were strongly induced in LMP2A-expressing cells (Figure 2B, lower three panels). These results thus demonstrate that LMP2A induces EMT-like molecular alterations in NPC cells. However, similar to a previously reported observation in squamous epithelial cells [35], LMP2A did not induce any obvious morphological changes in NPC cells in monolayer cultures.

To exclude the potential effects of selection, we then examined the representative EMT markers after transient tranfection of LMP2A in NPC cells. As shown in Figure S1, EMT-like molecular alterations were induced by transient expression of LMP2A in both CNE2 and SUNE1 cells. To further investigate whether endogenous LMP2A contributes to the EMT phenomenon, we tested whether NPC cells lacking this endogenous expression demonstrated any EMT-like cellular marker reversal as compared with LMP2A-expressing cells. Following the knockdown of LMP2A in C666 cells (Figure S2A), we found by immunofluorescence staining that the expression of the epithelial marker E-cadherin was up-regulated, whereas the mesenchymal marker vimentin was down-regulated on the membranes of the cells (Figure S2B). These results indicate that LMP2A is necessary for the EMT-like marker shift in NPC cells.

### LMP2A up-regulates stem cell marker expression and increases the stem cell-like population in NPC

**Figure 3:**
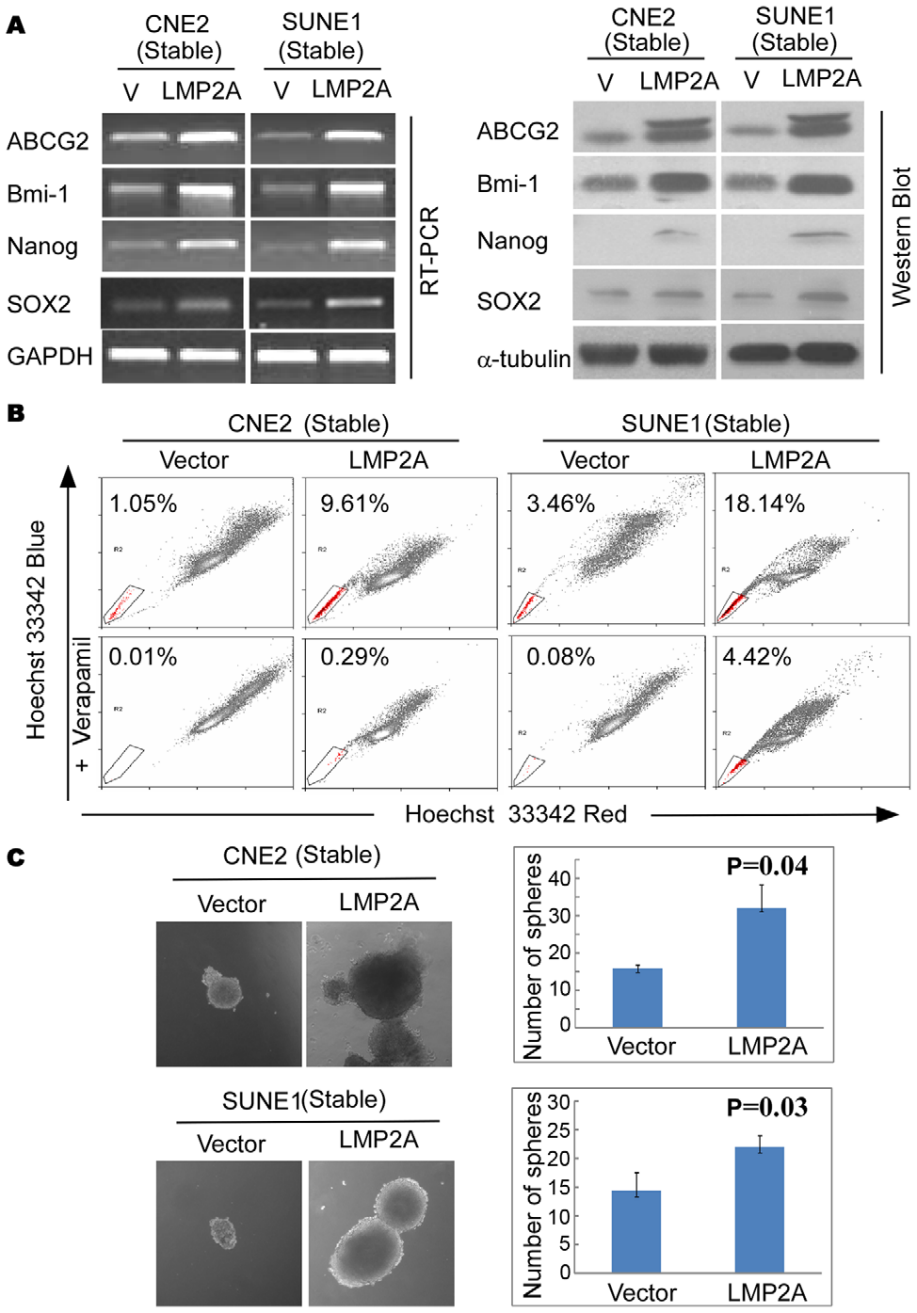
LMP2A induces stem-like properties in NPC cells. A. The right and left panels show up-regulated expression of the stem cell markers ABCG2, Bmi1, Nanog, SOX2 at both the mRNA and protein levels, respectively. B. LMP2A increases the size of the side population (SP) cells. Flow cytometric profiles of SP cells among the CNE2 and SUNE1 NPC cell lines after stable expression of LMP2A. SP cell profiles in the presence of verapamil are shown in the bottom panels. The percentages of SP cells are indicated. C. LMP2A induces stem cell-like self-renewal properties. Sphere sizes are shown in the left panels, and the numbers of spheres in both LMP2A-positive and -negative cells are shown in the right panels.

It has been reported recently that EMT generates cells showing the properties of stem cells [5]. We thus determined whether stable expression of LMP2A could induce such stem cell-like phenotypes in NPC. Representative stem cell markers were thus analyzed by RT-PCR or western blot. As shown in Figure 3A (left panel), in comparison with the vector control, LMP2A expression up-regulates the stem cell markers ABCG2, Bmi-1, Nanog, and SOX2 at the transcriptional level. The increases in ABCG2, Bmi-1, SOX2 and Nanog were further confirmed at the protein level (Figure 3A, right panel). As expected, transient expression of LMP2A could also induce stem cell markers, as demonstrated by the increased expression of ABCG2 and Bmi-1 at both transcriptional and protein levels (Figure S3A).

Side populations (SPs) among NPC cells have been reported to exhibit cancer stem cell characteristics [9]. We wished therefore to determine whether the increased expression of stem cell markers we observed in LMP2A-expressing cells was caused by an increase in the size of the stem cell-like SPs. As shown in Figure 3B, the stable expression of LMP2A dramatically increases the size of the SP in the CNE2 (from 1.04% to 8.32%) and SUNE1 (from 3.38% to 13.72%) cell lines. Importantly, SPs were also increased in transient LMP2A expressing cells CNE2 (from 1.56% to 3.65%) and SUNE1 (from 3.91% to 11.37%) (Figure S3B). Interestingly however, we did not observe any SPs in either wild type or LMP2A knockdown C666 cells.

Previously, we have reported that the side population (SP) cells, isolated from CNE2 NPC cell line, exhibited cancer stem cell characteristics [36]. Thus, we sorted the SP fraction in CNE2-Vector, CNE2-LMP2A, SUNE1-Vector and SUNE1-LMP2A cells, respectively, and then performed colony formation assay. As shown in Figure S4, SP fraction from either LMP2A or vector control cells form larger and more colonies compared with the non-SP fraction, confirmed that the stem cell population is indeed within the SP fraction in NPC cell lines. Taken together, our results demonstrate that LMP2A could induce expression of stem cell markers and increase the stem cell population in NPC cells.

### LMP2A induces stem cell-like self-renewal properties

We next analyzed whether the increase in the sizes of the SPs in NPC is due to the enhanced self-renewal properties of the stem-like cells therein. LMP2A and control cells were cultured in suspension to generate spheres, the number and sizes of which reflect both the quantity and ability of cells to self-renew *in vitro* [36]. As shown in Figure 3C, LMP2A-expressing cells formed more and larger spheres than vector controls cells did in both NPC cell lines (CNE2, *P* = 0.04; SUNE1, *P* = 0.03). We conclude from this that LMP2A can indeed enhance stem cell self-renewal properties, and thereby increase the size of these populations.

### LMP2A enhances the transforming ability of NPC cells

**Figure 4:**
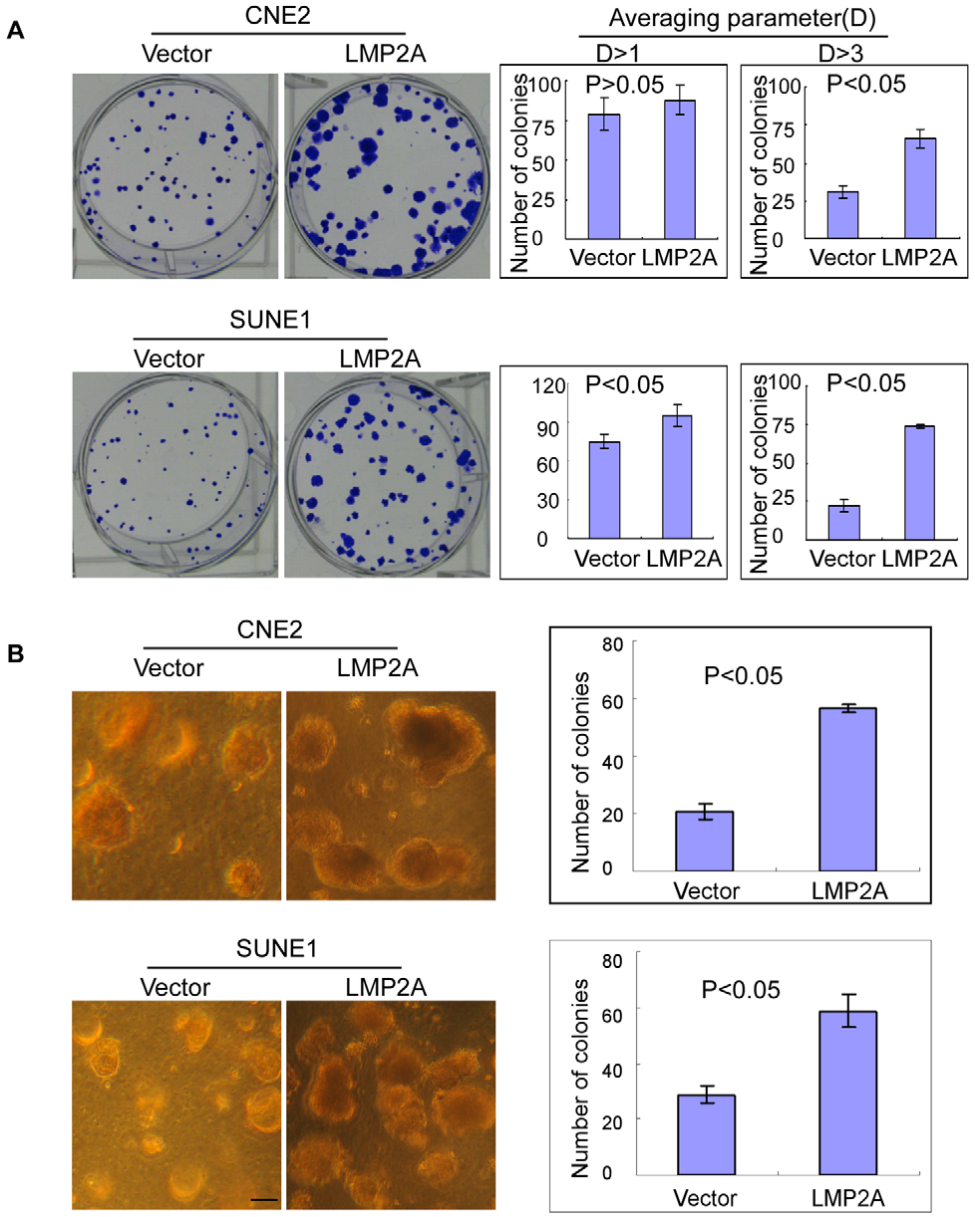
LMP2A enhances the transforming ability of NPC cells. A. Colony formation assay of the CNE2 and SUNE1 cell lines. Upon the stable expression of LMP2A, these cells form bigger (left panel) and more colonies (right panel) compared with the vector control cells. Error bar = SD. B. The anchorage-independent growth in soft agar of CNE2 and SUNE1 cells with or without exogenous LMP2A expression. Error bar = SD; scale bar = 200 µm.

To investigate whether LMP2A can enhance the transforming ability of NPC cells, we used both a colony formation and anchorage-independent growth assay in soft agar. We plated 200 NPC cells in triplicate wells of six-well plates for the colony formation assay. After 14 days of culture, LMP2A-expressing cells formed colonies that were significantly larger than those of the vector control cells (Figure 4A). There were also more LMP2A-expressing than vector control colonies. Statistical analysis showed significant differences in the number of colonies between the LMP2A-expressing and vector control cell lines (*P*<0.05; Figure 4A, right panel). In addition, the transforming ability of LMP2A expressing cells was also determined by soft agar assay. As shown in Figure 4B, LMP2A-expressing cells formed significantly more and larger colonies compared to the vector cells in soft agar assay.

### LMP2A increases the number of tumor initiating cells *in vivo*

**Figure 5:**
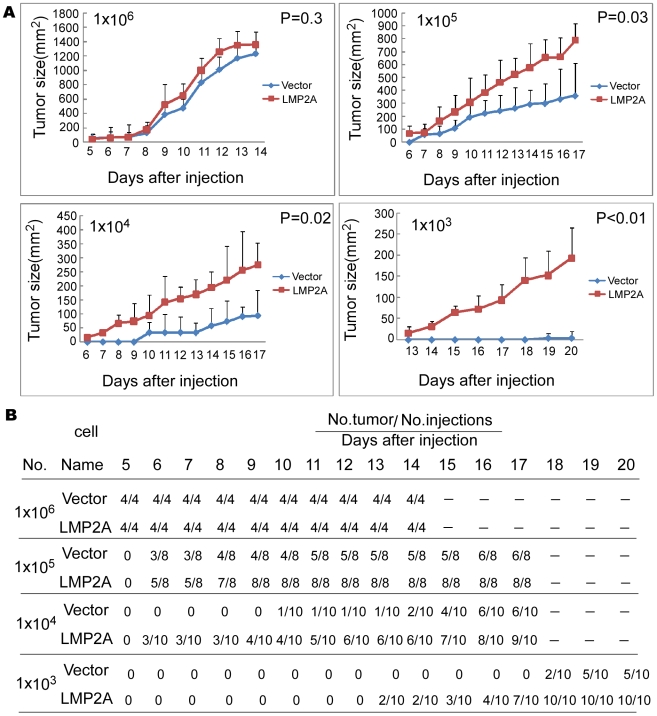
LMP2A enhances the initial tumor cell population in CNE2 cells. A. Tumor growth curves after injection of nude mice with LMP2A or vector control expressing NPC CNE2 cells. Once they become palpable, the LMP2A tumor (red) cells grow at a higher rate than the vector control (blue) cells in all cases. This difference becomes more pronounced when the injected cell number is 10^4^ and 10^3^. B. Tumors become palpable at an earlier timepoint and grow at a faster rate in LMP2A-expressing cells compared with vector control cells. Tumor formation was monitored for 20 days after injection of nude mice. Differences were not evident when the injected cell number was above 10^5^. However, at 10^3^ and 10^4^ cells, the differences were significant. In the case of injections with 10^4^ cells followed by monitoring for 17 days, 9 tumors arose in 10 mice for LMP2A-expressing cells, whereas only 6 of 10 mice infected with vector control cells formed tumors. When 10^3^ cells were injected followed by monitoring for 20 days, all mice formed tumors in the LMP2A-expressing cell group, but only half of the mice did so in the vector control group.

As SPs are enriched for tumor initiating cells, we next assessed the effects of LMP2A upon the tumorigenicity of NPC cell lines in nude mice. As shown in Figure 5, when injected with 1×10^6^ cells, the palpable tumors formed by LMP2A cells and control cells appeared at a similar time and grew at a comparable rate. As the injected cell number was reduced however (cell numbers at 1×10^5^, 1×10^4^ or 1×10^3^), the growth rates of the LMP2A tumors were found to be higher than those of controls injected with the same cell numbers. The data in Figure 5B show that when injected with 1×10^5^, 1×10^4^ or 1×10^3^ LMP2A-expressing NPC cells, 96% of the nude mice (27/28) developed tumors, whereas only 61% of these mice (17/28) did so when injected with the control cells. When 1×10^3^ cells were injected, the control cells formed only small tumors in 5/10 mice after 20 days whereas LMP2A-expressing cells formed tumors in 10/10 mice. In addition, the first palpable tumor in the LMP2A groups injected with 1×10^3^ cells appeared within 13 days, six days earlier than the control. Mice were sacrificed at 14, 17 or 20 days after injection, and the tumors were then weighed and photographed (Figure S5A and B). In all cases, the sizes of the tumors formed by the LMP2A-expressing NPC cells were larger than the vector control cells except in the 1×10^6^ cell inoculation groups. This difference was most apparent in the 1×10^3^ cell group (*P* = 0.004). Hence, LMP2A increases the number of tumor initiating cells in NPC.

### LMP2A expression correlates with EMT-like and stem cell markers' expression in NPC samples

**Figure 6:**
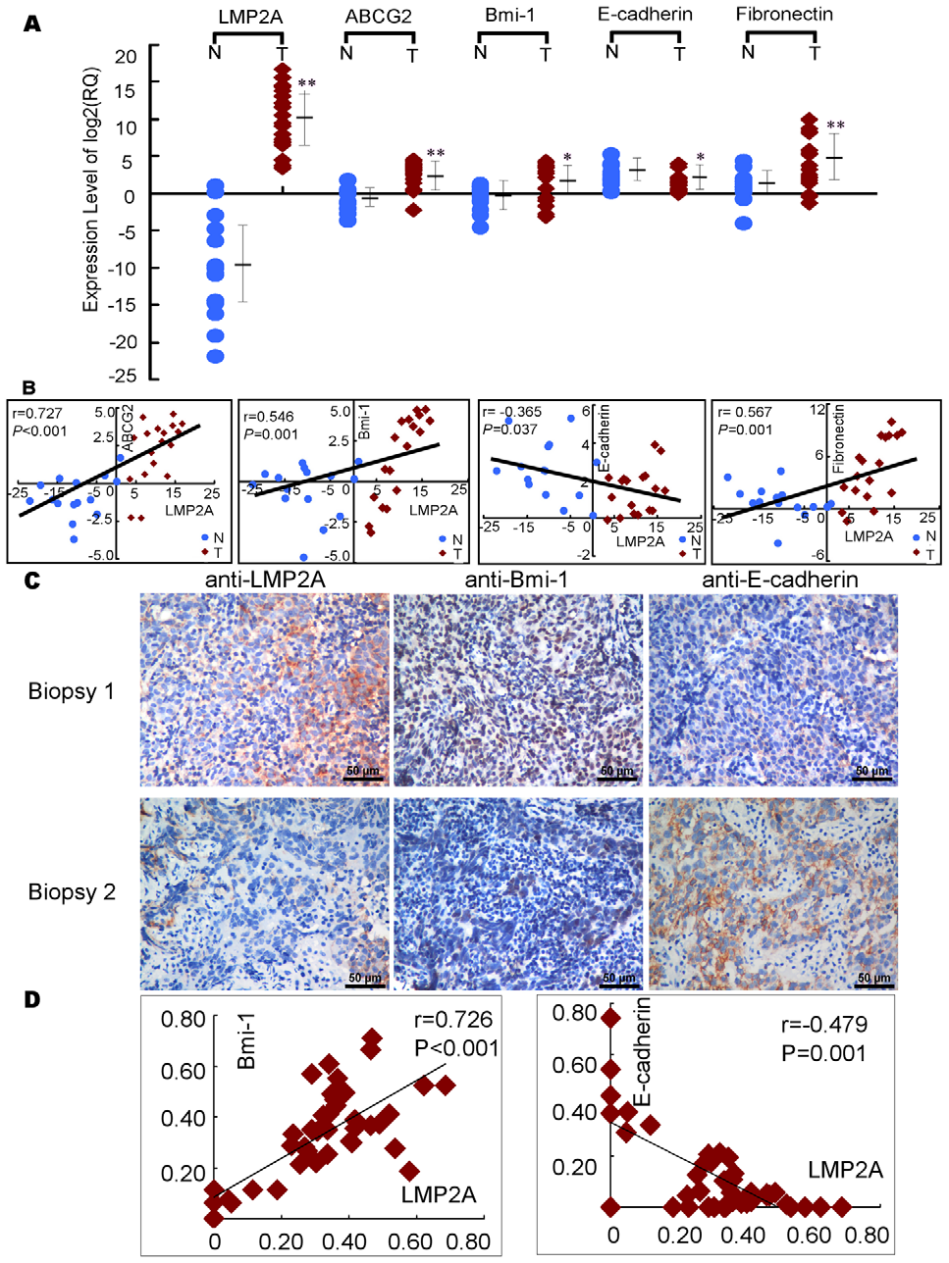
The expression of LMP2A correlates with the expression of EMT and stem cell-related markers in NPC. A. The mRNA level of LMP2A, ABCG2, Bmi-1, E-cadherin and Fibronectin in NPC biopsy samples and control inflammatory samples measured by real time RT-PCR. T = tumor, N = normal. B. LMP2A correlates positively with ABCG2, Bmi-1, Fibronectin, and negatively with E-cadherin in tissue samples. T = tumor, N = normal. C. The expression of LMP2A, Bmi-1 and E-cadherin in a cohort of NPC biopsy using immunohistochemistry assay. Final magnification 40X, scale bar = 50 µm. D. LMP2A significantly correlates with Bmi-1and E-cadherin in NPC tissues. The coordinate axis equals to the percentage of positive cells in whole cancer cells.

To determine whether any correlation existed between LMP2A expression and the representative markers of EMT and stem cell in NPC biopsy samples, we obtained RNA from 15 inflammatory samples and 18 NPC samples and analyzed LMP2A, ABCG2, Bmi-1, E-cadherin (E-cad) and Fibronectin (FN1) expression using real-time RT-PCR. LMP2A, Bmi-1 and ABCG2 transcripts were found to be low or undetectable in the 15 inflammatory samples but extremely high in the NPC tumor tissue (Figure 6A). We also found that LMP2A expression positively correlates with ABCG2, Bmi-1 and Fibronectin, and negatively correlates with E-cadherin (Figure 6B).

In addition, we also detected LMP2A, Bmi-1, E-cadherin proteins in another 42 NPC biopsies. As shown in Figure 6C and Figure 6D, LMP2A correlated positively with Bmi-1, and negatively with E-cadherin.

### Akt activity contributes to the up-regulation of SP cells by LMP2A

**Figure 7:**
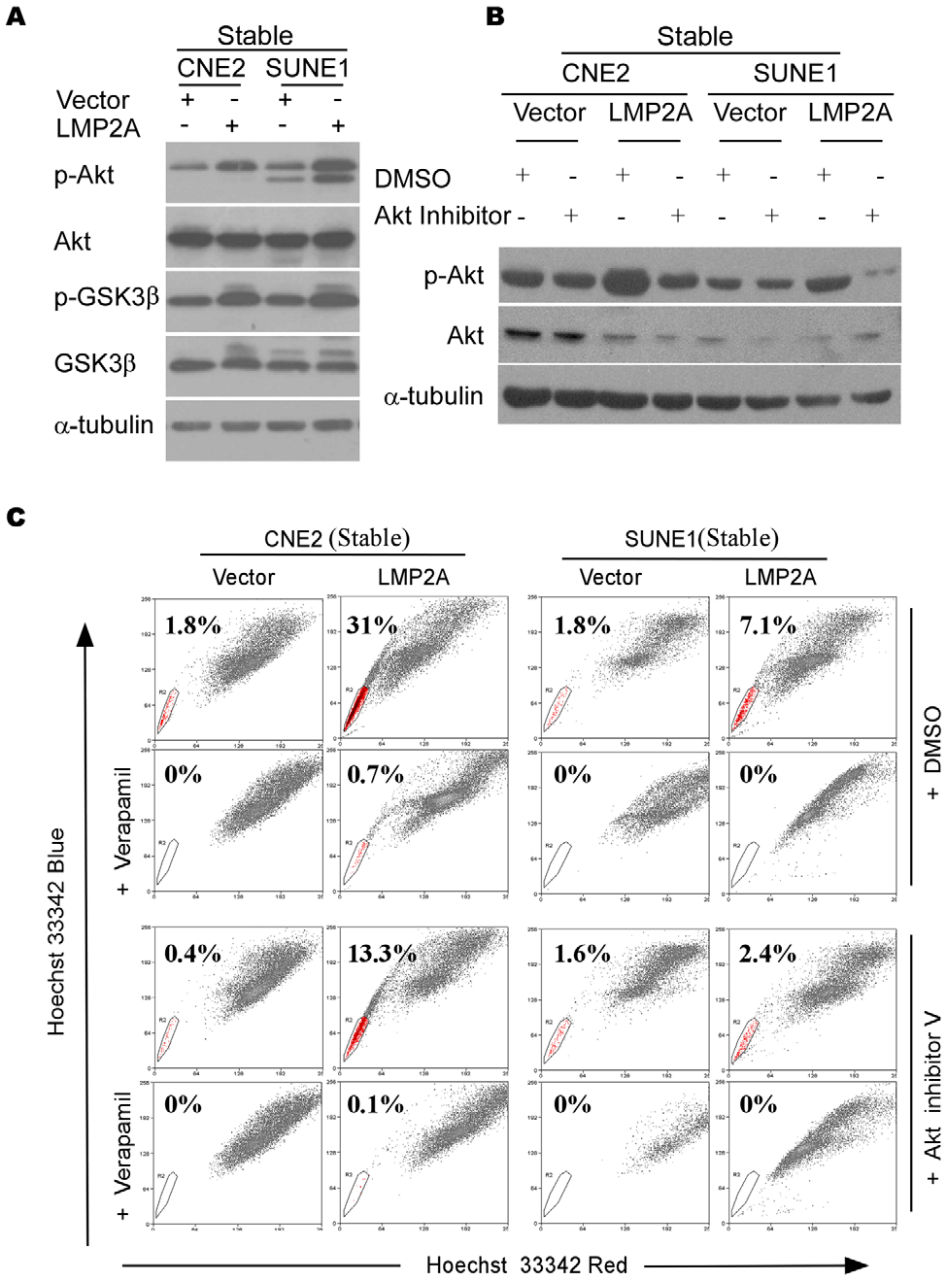
Akt contributes to the enhancement of the SP by LMP2A. A. Phospho-Akt (Thr308) and phospho-GSK3β were analyzed by Western blotting. B. The suppression of phospho-Akt (Thr308) in LMP2A-expressing and vector control cells after Akt inhibitor treatment determined by western blot analysis. C. The reduction in the SP after Akt inhibitor treatment detected by flow cytometry.

As previously shown, the expression of LMP2A in B lymphocytes and HaCaT cells induces the activation of Akt in a PI3K-dependent manner [26], [28]. To investigate the Akt status in NPC cells in our current study, western blot analysis using an antibody that detects Thr308 phosphorylation of Akt was performed to detect activated Akt (Figure 7A). Phospho-Akt (Thr308) was found to be up-regulated at least 2.5 folds in LMP2A-expressing cells compared with control cells as analyzed by Quantity One. Phospho-GSK3β, a direct target of Akt GSK3β [37], was further found to be induced in LMP2A cells (Figure 7A). After treatment with Akt inhibitor (V) at 4 µM for 12hours, the phosphorylation of Akt was suppressed in both the LMP2A and vector control NPC cells (Figure 7B). It is noteworthy, however, that the SPs were dramatically reduced in NPC cell lines in the presence of Akt inhibitor (V), particularly in LMP2A-expressing cells. As shown in Figure 7C, the size of the SP decreased from 31% to 13.3% in CNE2-LMP2A cells and from 7.1% to 2.4% in SUNE1-LMP2A cells. Thus, the Akt pathway seems to play a role in the LMP2A-mediated increase of NPC SP cells, although this will need to be further confirmed using dominant negative mutants or shRNAs that target Akt in LMP2A-expressing cells.

Most importantly, these results were further confirmed by transient expression of LMP2A. Figure S6A, phospho-Akt (Thr308) and phospho-GSK3β were upregulated in LMP2A positive cells consistent with the above results. And after treatment with Akt inhibitor (V), we observed the similar results (Figure S6B). Moreover, as shown in Figure S6C, the size of the SP decreased from 13% to 7.06% in transient CNE2-LMP2A cells.

## Discussion

The results of our current study indicate a pivotal role for LMP2A in the progression of NPC via stem-like cancer cell induction. The novel functions of LMP2A in inducing EMT, increasing the size of the SP, enhancing the self-renewal properties of stem-like cancer cells, and increasing the number of cancer initiating cells, confirm the involvement of this protein in oncogenic processes through the modulation of the CSC population in NPC.

Many studies have recently focused on the function of LMP2A in B cells. Although LMP2A is not required for EBV transformation of human B cells [38], [39], [40], it can drive B-cell development and survival by mimicking B-cell receptor (BCR) signal transduction [23], [41], [42], [43], [44]. The profiling of genes that are involved in different biological signaling pathways in EBV-associated cancers, in comparison with those of LMP2A transgenic mice, indicates that LMP2A may play a key role in tumorigenesis [45], [46]. As reported, LMP2A can be detected in approximately half of Hodgkin's lymphomas [47], and routinely detectable in nasopharyngeal carcinoma [19], [20], [21], suggesting that it may play important roles in the induction of human cancers of EBV. Previous studies suggesting that LMP2A may be involved in cell proliferation have come from the Raab-Traub and Tsai laboratories, which have independently demonstrated that LMP2A has dramatic effects on epithelial cells, such as the ability to accelerate anchorage-independent growth, promote tumor growth in nude mice, inhibit epithelial cell differentiation, and activate cell motility [24], [26].

Consistent with previous findings [20], [21], [22], we observed LMP2A protein in 57.6% of the NPC specimens analyzed in our present study. More importantly, we found that LMP2A localized predominantly at the tumor invasive front, indicating its role in promoting tumor invasion and migration. Indeed, invasion and metastasis are major features of NPC. Many types of cancer cells derived from primary carcinoma appear to rely on the EMT program to facilitate most of the steps in the invasion-metastasis cascade, in which the down-regulation of E-cadherin is a key initial event [48]. However, the last step in this process, which is termed colonization, requires that the cells that migrate from the original tumor site possess the self-renewal capability to form the macroscopic metastases in addition to an EMT ability [5]. The significance of our present results is underscored by the fact we have demonstrated for the first time that the EBV latent membrane protein LMP2A can confer stem-like properties upon NPC cells whilst at the same time promote EMT. Our findings are in agreement with and support the previous observation of a direct link between EMT and the gain of epithelial stem cell properties [49], to our knowledge the first report of a connection of this nature. It will be important to perform a study in a larger cohort of samples in the future to further demonstrate the correlation between LMP2A and ABCG2 expression and the clinicopathological characteristics of NPC.

Acquisition of the EMT phenotype in epithelial tumor cells is believed to play critical roles in the increased invasiveness and metastatic potential of tumor cells, and this process is causative for the development of “cancer stem-like cell” characteristics. The evidence thus far suggests that tumors arise from a diseased stem cell derived from a progenitor cell population and that many cancers, not unlike normal organs, contain a small population of cells with a high proliferative capacity, self-renewing potential, multi-differentiation ability, and that are resistant to chemotherapy and radiotherapy. All of these properties are characteristic of normal adult stem cells and even embryonic stem cells. Thus, this subpopulation of cells is denoted CSCs or tumor stem cells [50], [51], [52].

Our current study demonstrates that the LMP2A induces a stem cell state, evidenced by an enhanced self-renewal and transformational capacity, and also increases the number of tumor initiating cells *in vivo*. This was further confirmed by the greatly increased SP size and higher expression at both the transcriptional and translational level of some stem cell markers, such as ABCG2, Nanog, Bmi-1 and SOX2. Compared with most non-tumorigenic cancer cells, SP cells have a strong ability to form tumors after transplantation. Because SP cells are also resistant to chemotherapy and radiotherapy, they may contribute to tumor relapse even after most non-tumorigenic cells are destroyed [53]. Recent studies indeed show that the transcription factors OCT4, SOX2, and Nanog have essential roles in early development and are required for the propagation of undifferentiated embryonic stem (ES) cells in culture [54], [55], [56], [57]. More importantly, the positive correlation of LMP2A and ABCG2 expression in NPC specimens provides a valuable clue to further elucidating the processes underlying clinical metastasis and recurrence in NPC.

It is tempting to speculate regarding the actual mechanisms by which LMP2A induces EMT and cancer stem-like characteristics. Previous studies showed that Syk inhibition could impair LMP2A-mediated cell migration; and the mutation of Tyr-74 and Tyr-85, the LMP2A ITAM, simultaneously blocked Syk activation and cell migration [30]. It also has been previously shown that the constitutive activation of the Ras/PI3-K/Akt pathway by LMP2A is a key element of LMP2A-mediated transformation, whereas the cell adhesion signaling and MAPK pathways are not activated in LMP2A tumors [25], [26]. Our current data also show that LMP2A activates Akt in NPC cells and that after treatment with Akt inhibitor (V) for 12 hours, the SP cell population decreased greatly in LMP2A-expressing cells. It is well known that NF-κB plays an important role in mediating the processes of EMT induced by different factors through the upregulation of the transcriptional repressor functions of ZEB1 and ZEB2, the zinc-finger E-box binding homeobox proteins essential in E-cadherin regulation [58], [59]. Moreover, the PI3-K/Akt/mTOR pathway could indirectly activate NF-κB activity by regulating glycogen synthase kinase (GSK)-3β phosphorylation [60]. The importance of PI3K/Akt signaling in the proliferation and maintenance of embryonic stem cell (ESC) self-renewal has previously been suggested [33], [34], and its involvement in the regulation of the representative pluripotency marker genes, *Oct4*, *Sox2*, and *FoxD3*, has also been reported in a recent study [61]. Further more, LMP2A induces expression of polycomb group protein Bmi-1, which has been recently reported to play an important role in progression of NPC by inducing EMT and maintenance of stem-like phenotype via PTEN/Akt/Snail signaling [62]. Taken together, these results suggest that the PI3K/Akt pathway, at least in part, contributes to the EMT process and also the SP stem-like cancer cell shift of NPC epithelial cells. However, the underlying mechanisms how LMP2A modulates the Akt activity and expression of Bmi-1 in NPC cells requires further investigation. Importantly, some effects induced by LMP2A on EMT are also induced by LMP1 [63]. It will be interesting to determine whether LMP1 and LMP2A collaborate in inducing EMT and stemness of NPC cells.

It must be noted that the lack of effective SP sorting of endogenous LMP2A knockdown cells limits our ability to determine whether LMP2A alone is sufficient to increase the size of the SP. In addition, the complex regulatory machineries associated with LMP2A during cancer stem cell induction have not been fully elucidated. Hence, elucidation of the underlying signaling network that regulates the LMP2A pathways by using various LMP2A mutants and different pathway inhibitors will provide important further insights into the exact role of this viral protein in the emergence of cancer stem cells.

### Conclusions

We show for the first time herein that the EBV latent membrane protein LMP2A can induce EMT and increase the number of tumor initiating cells. Our data first indicated that LMP2A strongly up-regulates the cancer stem cell-like population in NPC, which may explain the onset of metastases and high rate of recurrence for these tumors. This raises the possibility that this viral protein plays a key role not only in EBV latency and persistence but also in the progression of NPC. Based on our novel findings, we believe that the pathologic diagnosis together with detection of LMP2A in tumor tissue will aid in predicting NPC progression, and that LMP2A can be considered to be a novel therapeutic target for this cancer.

## Materials and Methods

### Ethics statement

All animal work was conducted under the institutional guidelines of Guangdong Province and approved by the Use Committee for Animal Care. Approval from the Sun Yat-sen University Institute Research Ethics Committee was obtained, and written informed consent was provided by each human subject.

### Establishment of LMP2A stable expressing and knockdown cell lines

Two poorly differentiated nasopharyngeal carcinoma cell lines (CNE2, SUNE1) were maintained in RPMI 1640 medium (Life Technologies, Carlsbad, CA) supplemented with 10% fetal bovine serum (FBS) in a humidified 5% CO_2_ incubator at 37°C. To generate stable cell lines, recombinant retroviruses expressing either vector pBabe or pBabe subcloned with LMP2A were generated as previously described [64] and used to infect CNE2 and SUNE1 cells [65]. Pooled CNE2 and SUNE1 cell populations expressing either pBabe or pBabe-LMP2A were selected with 0.5 µg/mL of puromycin (Sigma-Aldrich, St Louis, MO).

C666, the only well-known nasopharyngeal carcinoma cell line consistently carrying EBV, was chosen to perform the stable knockdown of LMP2A expression. Retroviral particles were generated and used to infect the target C666 cells as described previously [66]. The successful knockdown of LMP2A was verified by RT-PCR and immunofluorescence.

### Antibodies

An LMP2A monoclonal antibody was obtained from Proteintech Group Inc. ABCG2 (Cat. 3380) and Nanog (Cat. 21603) antibodies were obtained from Abcam (Cambridge, UK). Antibodies raised against E-cadherin (Cat. 610181), α-catenin (Cat. 610193), fibronectin (Cat. 610077), and vimentin (Cat. 550513) were purchased from BD Biosciences (Franklin Lakes, NJ). Mouse anti-Bmi-1 (Upstate Biotechnology, Lake Placid, NY), and rabbit-anti-GSK-3β, p-GSK-3β, Akt (Cell Signaling, Beverly, MA) and p-Akt (Santa Cruz Biotechnology, CA.) primary antibodies, and FITC or rhodamine-conjugated goat anti-rabbit IgG or goat anti-mouse IgG (Jackson Laboratory, West Grove, PA) or Peroxidase-conjugated goat anti-rabbit IgG or goat anti-mouse IgG (Amersham Pharmacia Biotech, Piscataway, NJ) secondary antibodies were used for western blot or immunofluorescence analysis.

### Tissue samples

Freshly frozen biopsied tissues from a total of 18 NPC patients and 15 normal controls, and 81 paraffin-embedded NPC samples which had been histologically and clinically diagnosed were collected from the archives of the Department of Sample Resources, Cancer Center, Sun Yat-sen University (Guangzhou, China). Prior informed consent from the patients and approval from the Institute Research Ethics Committee was obtained.

### Sorting of SP cells by flow cytometry

Cells were analyzed by FACS when the cells had reached a logarithmic growth phase (24 hours after replating). Cells were digested with 0.25% trypsin (Sigma-Aldrich, St. Louis, MO), washed twice with calcium/magnesium-free PBS, resuspended in ice-cold RPMI 1640 culture (supplemented with 2% FBS) at a concentration of 1×10^6^ cells/mL, and incubated at 37°C in a 5% CO_2_ incubator for 10 min. The DNA binding dye, Hoechst 33342 (Sigma-Aldrich, St. Louis, MO), was then added at a final concentration of 5 µg/mL and the samples were incubated for 90 min in the dark with periodic mixing. The cells were then washed twice with PBS, 1 µg/mL propidium iodide (Sigma-Aldrich) was added, and the cells were kept at 4°C in dark prior to sorting by a Moflo XDP (Beckman Coulter, Fullerton, CA). Because Hoechst 33342 extrudes from cells treated with verapamil (a calcium ion tunnel antagonist)-sensitive ABC transporters, a subset of the cells were incubated with 50 µmol/L verapamil for 30 min at 37°C before the addition of Hoechst 33342 to determine whether this would block the fluorescent efflux of SP cells in the CNE2 and SUNE1 populations.

### RT-PCR

Total RNA extracts from LMP2A-overexpressing cells and pBabe vector control cells were prepared using a Trizol reagent (Life Technologies, Grand Island, NY) according to the manufacturer's instructions. The RNA was then treated with DNase, and 2.5 µg aliquots were used for cDNA synthesis using random hexamers. The primers used for the amplification of the indicated genes are listed in Table S1.

### Real time RT-PCR

The expression levels of LMP2A, ABCG2, BMI-1, E-cadherin and Fibronectin mRNA was determined by SYBR green real-time reverse transcription-PCR (RT-PCR). Total RNA from different human nasopharyngeal tissues were extracted using Trizol reagent (Invitrogen, Carlsbad, CA). Quantitative dertermination of RNA levels were performed in triplicate in three independent experiments. Real-time PCR and data collection were performed with an ABI PRISM 7900HT sequence detection system. The housekeeping gene GAPDH was used as an internal control to normalize the expression levels of different genes. The primers used for the amplification of the indicated genes are listed in Table S2.

### Western blotting

Western blotting analysis was performed as previously described [67]. Where relevant, the blots were probed with antibodies as labeled in the figures, and the signals were detected using enhanced chemiluminescence (ECL) (Amersham Pharmacia Biotech, Piscataway, NJ). The membranes were stripped and probed with an anti-alpha tubulin mouse monoclonal antibody (Santa Cruz Biotechnology, Santa Cruz, CA) to confirm equal loading of the samples.

### Immunofluorescence analysis

Immunofluorescence analysis was performed as described previously [67]. Cell lines were plated on culture slides (Costar, Cambridge, MA) and after 24 hours were rinsed with phosphate-buffered saline (PBS) and fixed in ice-cold methanol-acetone for 5 min at -20°C. The cells were then blocked for 30 min in 10% BSA (Sigma-Aldrich St. Louis, MO) in PBS and then incubated with primary monoclonal antibodies in PBS for 2 hours at room temperature. After three washes in PBS, the slides were incubated for 1 h in the dark with secondary goat anti-mouse, or goat anti-rabbit antibodies (Invitrogen, Carlsbad, CA). After three further washes, the slides were stained with 4-,6-diamidino-2-phenylindole (DAPI; Sigma-Aldrich St. Louis, MO) for 5 min to visualize the nuclei, and examined using an Olympus confocal imaging system (Olympus FV100).

### Anchorage-independent growth assay

Six-well plates were coated with a layer of 0.6% agar in medium supplemented with 20% fetal bovine serum. Cells were prepared in 0.3% agar and seeded in triplicate. The plates were then incubated at 37°C in a humid atmosphere of 5% CO_2_ for two weeks until colonies had formed. Each experiment was repeated at least three times. Colonies were photographed between 18–24 days (final magnification 20 X) under a phase contrast microscope, and colonies larger than 50 µm in diameter were counted under a light microscope.

### Colony formation assay

Cells were counted, plated in triplicate at 200 cells for the pooled population or 100 sorted cells per well in six-well plates, and cultured with RPMI 1640 complete culture for 10 days. After most of the colonies had expanded to more than 50 cells, they were washed twice with PBS, fixed in methanol for 15 min, and dyed with crystal violet for 15 min at room temperature. After washing out the dye, the plates were photographed. To quantify the colonies objectively, the software Quantity One was used and colonies that lager than the averaging parameter of 3 or 1 and the minimum signal intensity of 1.0 were counted. At least three independent experiments were carried out for each assay.

### Tumor formation in an animal model

Nude mice were purchased from the Shanghai Slac Laboratory Animal Co. Ltd and maintained in microisolator cages. All animals were used in accordance with institutional guidelines and the current experiments were approved by the Use Committee for Animal Care. Tumor cells were suspended in 200 µl RPMI 1640 complete culture with 25% Matrigel (BD Biosciences) and inoculated subcutaneously into the left flanks of 4- to 5-week-old nude mice. The mice were monitored daily for palpable tumor formation and tumors were measured using a Vernier caliper, and also weighed and photographed.

### Accession numbers

The Entrez Gene ID for genes and proteins mentioned in the text are 3783751 (LMP2A), 2597 (GAPDH), 10376 (α-Tubulin), 999 (E-cadherin), 2335 (Fibronectin), 7431(Vimentin), 1495 (α-Catenin), 9429 (ABCG2), 648 (Bmi-1), 79923 (Nanog), 6657 (SOX2), NG_012188 (Akt), NG_012922 (GSK-3β).

## Supporting Information

Table S1 Primers for RT-PCR
(0.03 MB DOC)

Table S2 Primers for real-time RT-PCR
(0.03 MB DOC)

Figure S1 Transient expression of LMP2A induces EMT molecular alterations. 5×10^5^ CNE2 or SUNE1 cells were seeded per 100-mm dish and transfected next day with 8 µg of pCR3.1-LMP2A or control vector together with 1µg of EGFP-expressing vector using the FuGENE 6 reagent. EGFP positive cells were sorted out from the co-transfected cells 48h later, and then were used for western blot (A) or immunostaining analysis (B) to detect the EMT related markers as indicated. The expression of LMP2A in the majority of the sorted cells was confirmed by immunofluorescence staining (B, upper panel).
(3.43 MB TIF)

Figure S2 The suppression of endogenous LMP2A reverses the EMT-like cellular marker shift. A. Stable knockdown of endogenous LMP2A in EBV-positive NPC (C666) cells verified by immunofluorescence staining (left panel) and RT-PCR (right panel). B. The expression of E-cadherin and vimentin in LMP2A-shRNA and control C666 cells analyzed by immunofluorescence staining.
(4.81 MB TIF)

Figure S3 LMP2A up-regulates stem cell marker expression (A) and increases the stem cell-like population (B) in transiently transfected NPC cell lines. A. Cells were transfected and sorted as described in Figure S1. The sorted cells were used for detection the representative stem cell markers ABCG2 and Bmi-1 at both the mRNA (left panel) and protein levels (right panel). B. 5×10^5^ CNE2 or SUNE1 cells were seeded per 100mm dish and transfected next day with 8 µg of pCR3.1-LMP2A or control vector. The transfected cells were replated 24h later, and were cultured for another 24h before SP fraction analysis. SP cell profiles in the presence of verapamil are shown in the bottom panels. The percentages of SP cells are indicated.
(0.65 MB TIF)

Figure S4 SP fraction cells present high colony formation ability. Colony formation assay of the non-SP fraction and SP fraction from CNE2 vector cells (A), CNE2-LMP2A cells (B), SUNE1 vector cells (C), SUNE1-LMP2A cells (D). The SP fraction from either LMP2A or vector control cells form larger (left panel) and more colonies (right panel) compared with the non-SP fraction. 100 sorted SP or non-SP fraction cells were seeded per well in six-well plates and cultured for 10 days. The representative results of three independent experiments were presented as indicated. Error bar = SD.
(5.68 MB TIF)

Figure S5 LMP2A increases the tumor initiating cell number in CNE2 cells in vivo. A. NPC CNE2 tumors were weighed and photographed after dissection from nude mouse xenografts. In all cases, the tumors formed by LMP2A-expressing cells were larger than the controls. This difference was most pronounced for injections with 10^3^ cells. B. Statistical analysis of NPC CNE2 tumor formation. Following injection with 10^5^ and 10^6^ cells, no significant differences were evident. At 10^4^ cells however, the tumors formed by LMP2A-expressing cells were much larger than those derived from vector control cell injections although this was not statistically significant. Following 10^3^ cell injections however, the differences were statistically significant (*P*<0.01).
(3.26 MB TIF)

Figure S6 Akt activity contributes to the up-regulation of SP cells in transiently tranfected LMP2A cells. A, B. Cells were co-transfected and sorted as described in Figure S1. The sorted GFP positive cells were either used to analyze the phospho-Akt (Thr308) and phospho-GSK3β by western blotting (A), or replated and harvested for analysis of phospho-Akt (Thr308) after after Akt inhibitor treatment (B). C. Cells were transfected and processed in a similar way as described in Figure S2B, in addition treated by Akt inhibitor treatment for 8h before SP analysis.
(0.93 MB TIF)
