# Supplementary material for: Epstein-Barr Virus-Encoded LMP2A Induces an Epithelial–Mesenchymal Transition and Increases the Number of Side Population Stem-like Cancer Cells in Nasopharyngeal Carcinoma
Source: PLoS Pathog. 2010 Jun 3;6(6):e1000940. doi: 10.1371/journal.ppat.1000940 (PMC2880580; doi:10.1371/journal.ppat.1000940)
Supplement: Table S1 — Primers for RT-PCR (0.03 MB DOC) [file ppat.1000940.s001.doc]

**Table S1. Primers for RT-PCR**

| ABCG2 | Forward: 5’-GGGTTCTCTTCTTCCTGACGACC-3’  Reverse: 5’-TGGTTGTGAGATTGACCAACAGACC-3’ |
| --- | --- |
| SOX2 | Forward: 5’-AGCAACGGCAGCTACAGCA-3’  Reverse: 5’-TGGGAGGAAGAGGTAACCACAG-3’ |
| Nanog | Forward: 5’-CCTATGCCTGTGATTTGT-3’  Reverse: 5’-TGTTGACCGGCTTCTTAT-3’ |
| Bmi1 | Forward: 5’-ATGCATCGAACAACGAGA-3’  Reverse: 5’-TCAACCAGAAGAAGTTGCTG-3’ |
| β-actin | Forward: 5’-GAGTCAACGGATTTGGTCGT-3’  Reverse: 5’-GACAAGCTTCCCGTTCTCAG-3’ |
