# Supplementary material for: Epstein-Barr Virus-Encoded LMP2A Induces an Epithelial–Mesenchymal Transition and Increases the Number of Side Population Stem-like Cancer Cells in Nasopharyngeal Carcinoma
Source: PLoS Pathog. 2010 Jun 3;6(6):e1000940. doi: 10.1371/journal.ppat.1000940 (PMC2880580; doi:10.1371/journal.ppat.1000940)
Supplement: Table S2 — Primers for real-time RT-PCR (0.03 MB DOC) [file ppat.1000940.s002.doc]

**Table S2. Primers for real-time RT-PCR**

| ABCG2 | Forward: 5’-GCTTATTCAGCCAGTTCCAT-3’  Reverse: 5’-ATGCAATttgGGTGAGATTG-3’ |
| --- | --- |
| Bmi-1 | Forward: 5’- CTGGTTGCCCATTGACAGC-3’  Reverse: 5’- CAGAAAATGAATGCGAGCCA-3’ |
| E-cadherin | Forward: 5’- CTGCCCAGAAAATGAAAAAGG-3’  Reverse: 5’- AGTGTATGTGGCAATGCGTTC-3’ |
| Fibronectin | Forward: 5’- CAGTGGGAGACCTCGAGAAGA-3’  Reverse: 5’- GTCCCTCGGAACATCAGAAAC-3’ |
| LMP2A | Forward: 5’-TGCAATTTGCCTAACATGGA-3’  Reverse: 5’-GAGCACAAGCATCACCAGGA-3’ |
