# Supplementary figures and images for: Epstein-Barr Virus-Encoded LMP2A Induces an Epithelial–Mesenchymal Transition and Increases the Number of Side Population Stem-like Cancer Cells in Nasopharyngeal Carcinoma
Source: PLoS Pathog. 2010 Jun 3;6(6):e1000940. doi: 10.1371/journal.ppat.1000940 (PMC2880580; doi:10.1371/journal.ppat.1000940)

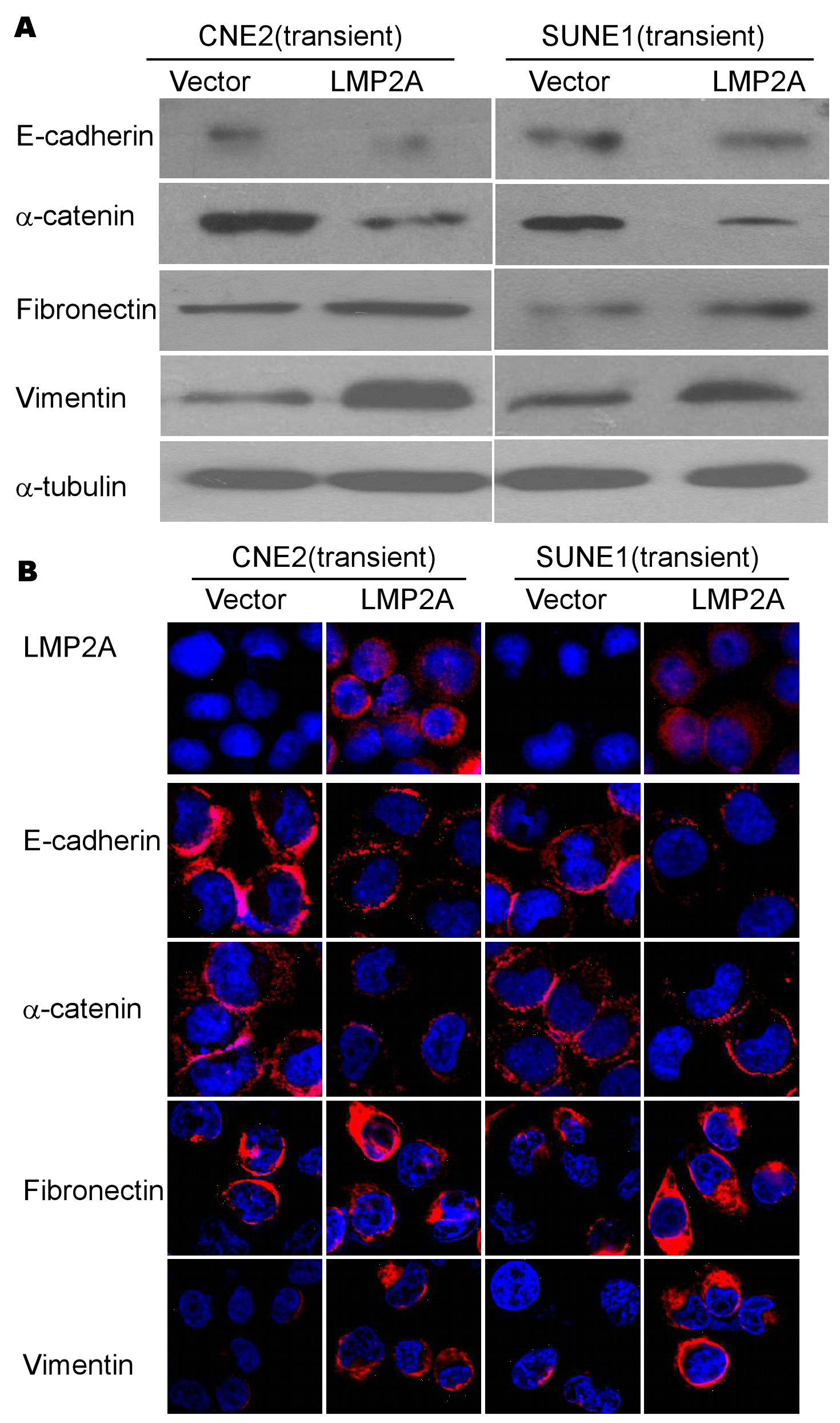

Supplement: Figure S1 — Transient expression of LMP2A induces EMT molecular alterations. 5×10^5^ CNE2 or SUNE1 cells were seeded per 100-mm dish and transfected next day with 8 µg of pCR3.1-LMP2A or control vector together with 1µg of EGFP-expressing vector using the FuGENE 6 reagent. EGFP positive cells were sorted out from the co-transfected cells 48h later, and then were used for western blot (A) or immunostaining analysis (B) to detect the EMT related markers as indicated. The expression of LMP2A in the majority of the sorted cells was confirmed by immunofluorescence staining (B, upper panel). (3.43 MB TIF) [file ppat.1000940.s003.tif]

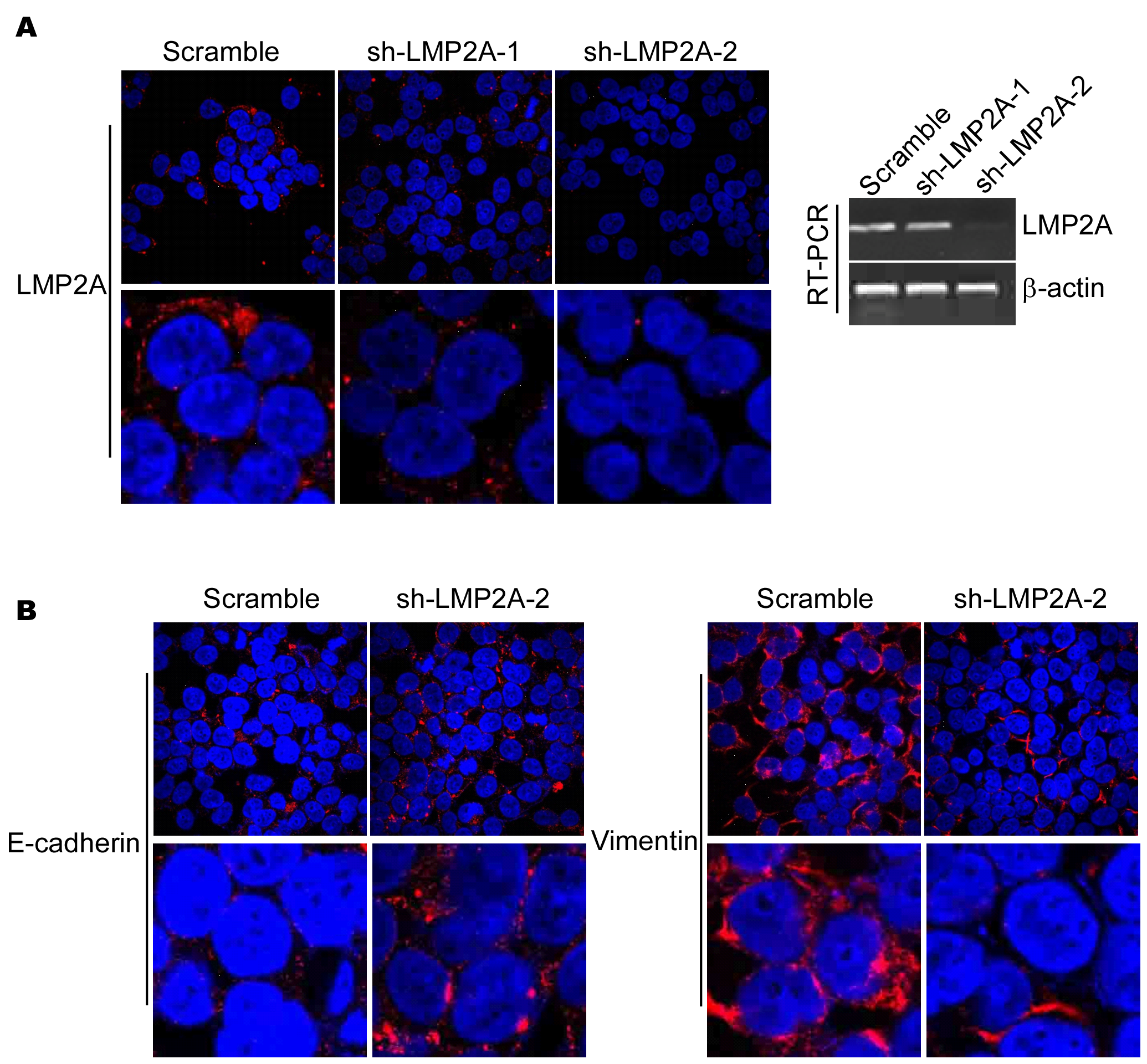

Supplement: Figure S2 — The suppression of endogenous LMP2A reverses the EMT-like cellular marker shift. A. Stable knockdown of endogenous LMP2A in EBV-positive NPC (C666) cells verified by immunofluorescence staining (left panel) and RT-PCR (right panel). B. The expression of E-cadherin and vimentin in LMP2A-shRNA and control C666 cells analyzed by immunofluorescence staining. (4.81 MB TIF) [file ppat.1000940.s004.tif]

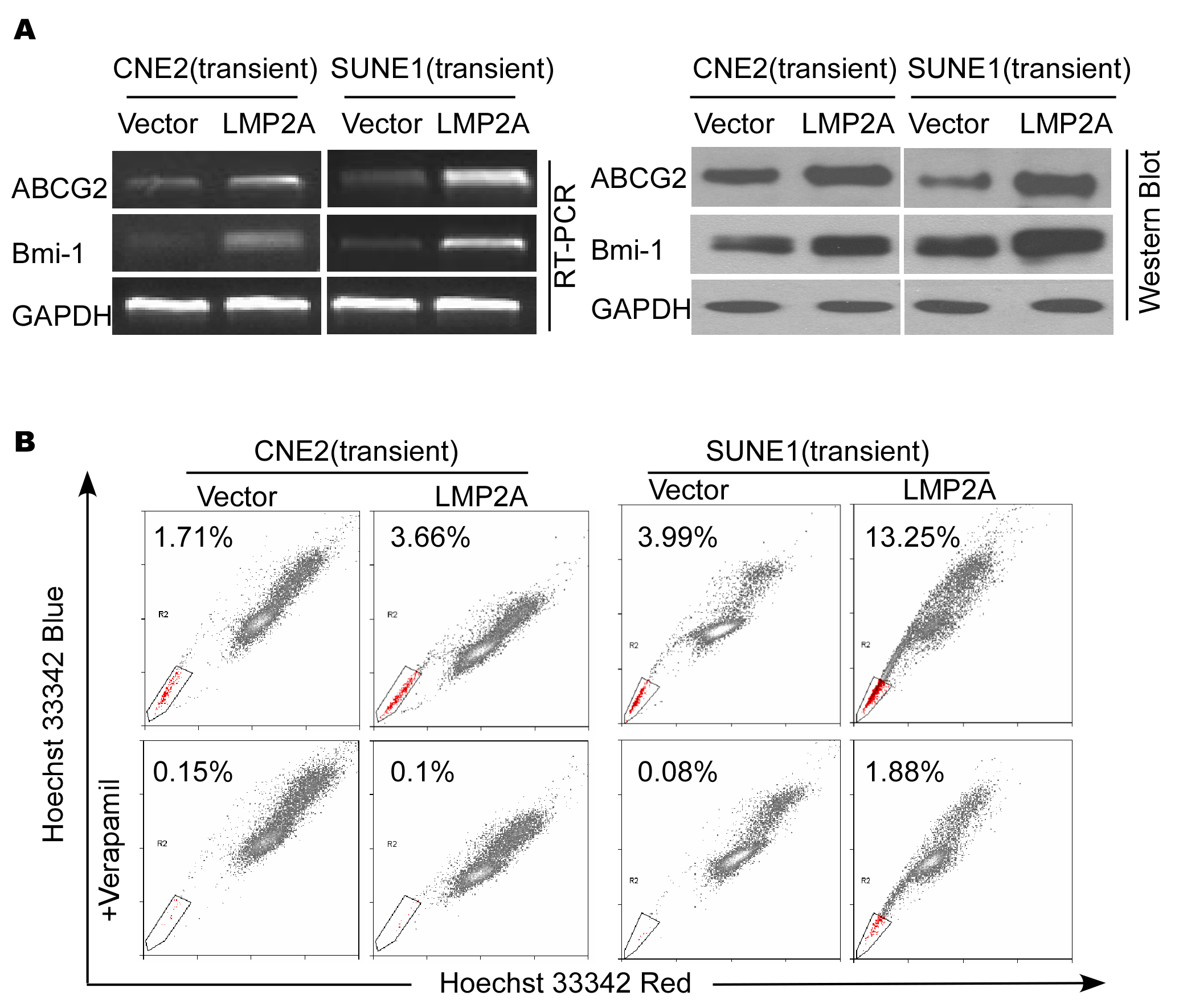

Supplement: Figure S3 — LMP2A up-regulates stem cell marker expression (A) and increases the stem cell-like population (B) in transiently transfected NPC cell lines. A. Cells were transfected and sorted as described in Figure S1. The sorted cells were used for detection the representative stem cell markers ABCG2 and Bmi-1 at both the mRNA (left panel) and protein levels (right panel). B. 5×10^5^ CNE2 or SUNE1 cells were seeded per 100mm dish and transfected next day with 8 µg of pCR3.1-LMP2A or control vector. The transfected cells were replated 24h later, and were cultured for another 24h before SP fraction analysis. SP cell profiles in the presence of verapamil are shown in the bottom panels. The percentages of SP cells are indicated. (0.65 MB TIF) [file ppat.1000940.s005.tif]

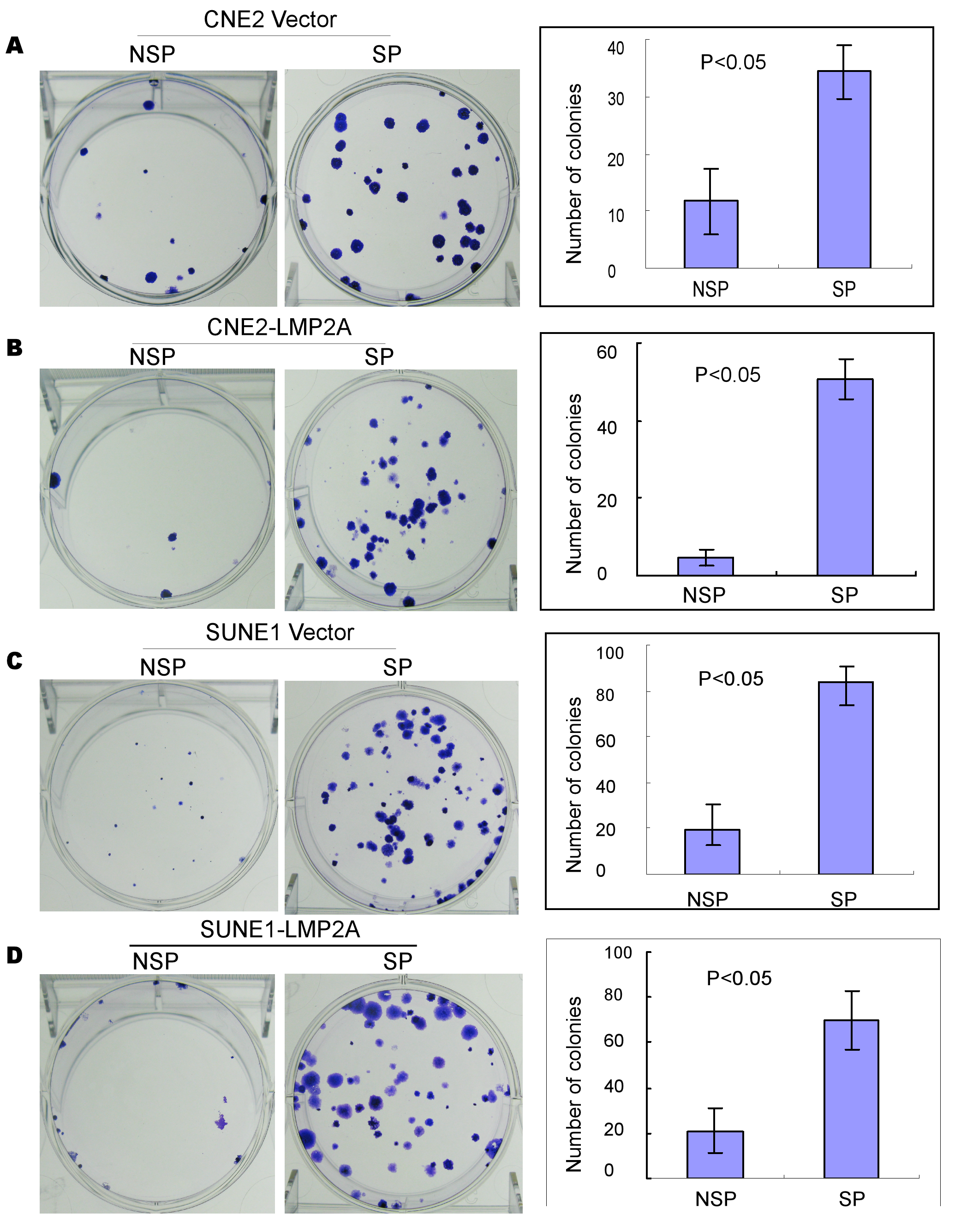

Supplement: Figure S4 — SP fraction cells present high colony formation ability. Colony formation assay of the non-SP fraction and SP fraction from CNE2 vector cells (A), CNE2-LMP2A cells (B), SUNE1 vector cells (C), SUNE1-LMP2A cells (D). The SP fraction from either LMP2A or vector control cells form larger (left panel) and more colonies (right panel) compared with the non-SP fraction. 100 sorted SP or non-SP fraction cells were seeded per well in six-well plates and cultured for 10 days. The representative results of three independent experiments were presented as indicated. Error bar = SD. (5.68 MB TIF) [file ppat.1000940.s006.tif]

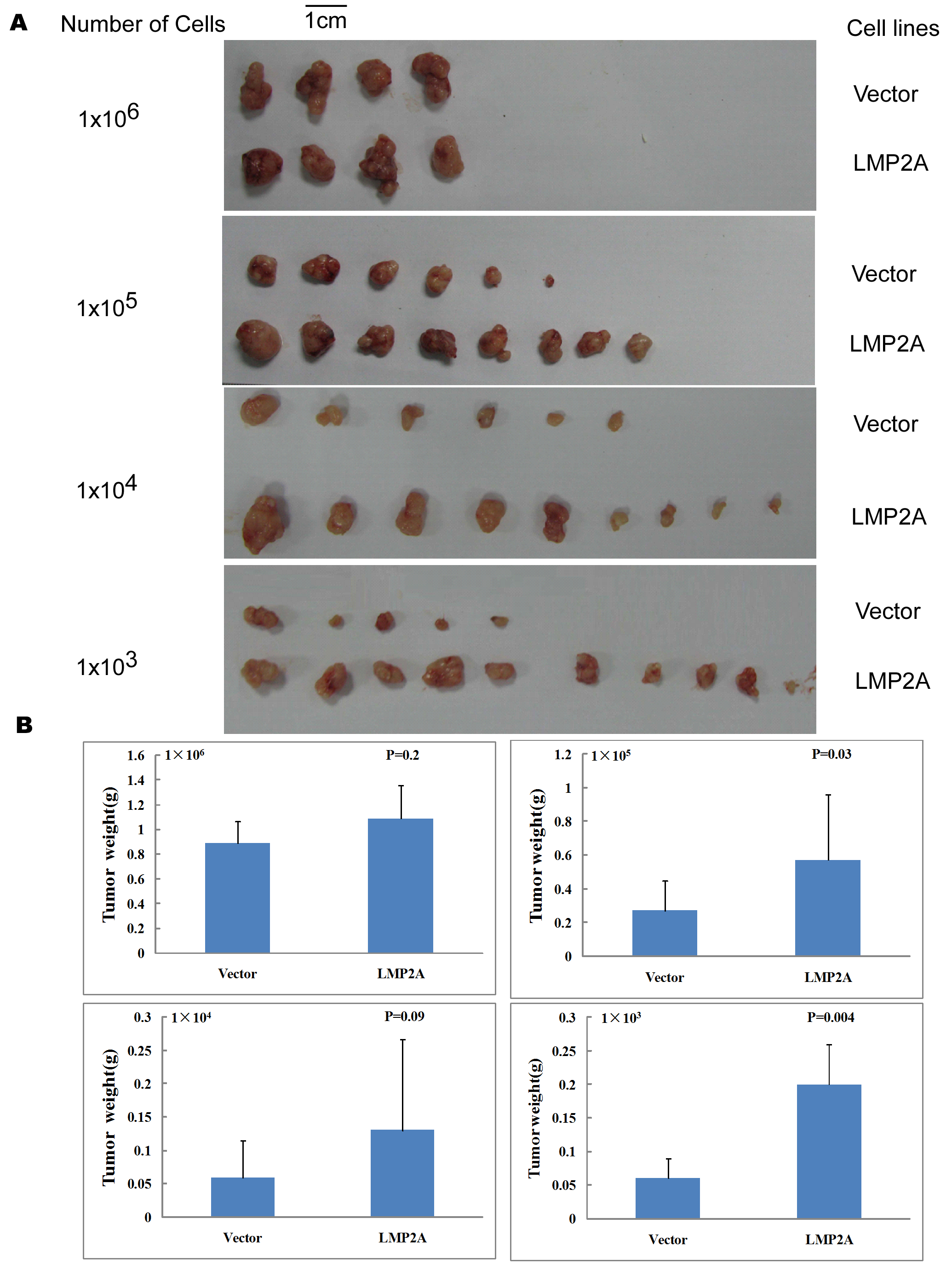

Supplement: Figure S5 — LMP2A increases the tumor initiating cell number in CNE2 cells in vivo. A. NPC CNE2 tumors were weighed and photographed after dissection from nude mouse xenografts. In all cases, the tumors formed by LMP2A-expressing cells were larger than the controls. This difference was most pronounced for injections with 103 cells. B. Statistical analysis of NPC CNE2 tumor formation. Following injection with 105 and 106 cells, no significant differences were evident. At 104 cells however, the tumors formed by LMP2A-expressing cells were much larger than those derived from vector control cell injections although this was not statistically significant. Following 103 cell injections however, the differences were statistically significant (P<0.01). (3.26 MB TIF) [file ppat.1000940.s007.tif]

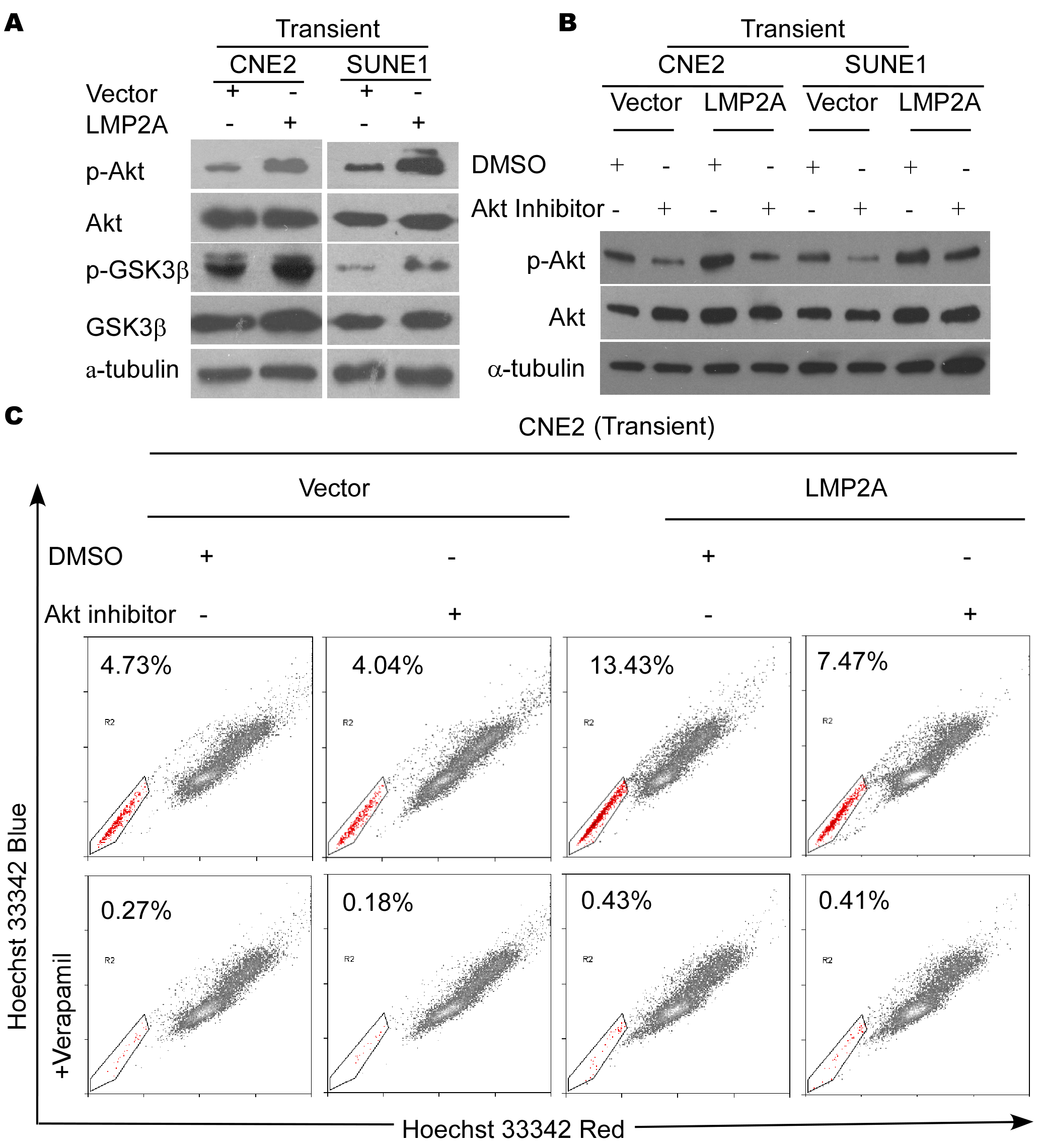

Supplement: Figure S6 — Akt activity contributes to the up-regulation of SP cells in transiently tranfected LMP2A cells. A, B. Cells were co-transfected and sorted as described in Figure S1. The sorted GFP positive cells were either used to analyze the phospho-Akt (Thr308) and phospho-GSK3β by western blotting (A), or replated and harvested for analysis of phospho-Akt (Thr308) after after Akt inhibitor treatment (B). C. Cells were transfected and processed in a similar way as described in Figure S2B, in addition treated by Akt inhibitor treatment for 8h before SP analysis. (0.93 MB TIF) [file ppat.1000940.s008.tif]
